# Case Report: Hindlimb Ataxia Concurrent With Seizures by Presumed Osmotic Demyelination Syndrome in a Dog

**DOI:** 10.3389/fvets.2022.848405

**Published:** 2022-06-17

**Authors:** Ga-Won Lee, Min-Hee Kang, Hee-Myung Park

**Affiliations:** ^1^Laboratory of Veterinary Internal Medicine, College of Veterinary Medicine, Konkuk University, Seoul, South Korea; ^2^Department of Bio-Animal Care, Jangan University, Suwon, South Korea

**Keywords:** ataxia, dog, osmotic demyelination syndrome, seizure, myelinolysis

## Abstract

A 6-year-old castrated male Chihuahua dog was presented with hindlimb paresis and ataxia. The dog had hyponatremia and was diagnosed as hypoadrenocorticism 10 days before its visit, and the neurologic signs including generalized tonic seizures and hindlimb paresis occurred 3 days after correction of hyponatremia at a referral hospital. Based on history and clinical findings, osmotic demyelination syndrome (ODS) secondary to rapid correction of hyponatremia was highly suspected. After administration of anti-convulsant and supplements, seizures did not occur, and gait was normalized within 2 weeks. Phenobarbital was tapered and finally discontinued after 3 months, and seizure did not recur. The neurologic signs were completely resolved and the dog continued to be free of neurologic or additional clinical signs over the 19-month follow-up period. ODS should be included among the differential diagnoses in case of any acute neurological dysfunction that occurs with episodes of rapid correction of hyponatremia. To the author's knowledge, this is the rare case report of a dog with hypoadrenocorticism and presumed ODS after rapid correction of hyponatremia leading to neurologic signs including seizures and ataxia.

## Introduction

Hypoadrenocorticism is a relatively uncommon endocrinopathy in dogs and even more rare in cats, resulting from an inability of the adrenal glands to produce or secrete adequate amounts of cortisol (1). The most common cause of hypoadrenocorticism is primary dysfunction from immune-mediated destruction of the adrenal cortex, which results in glucocorticoid and mineralocorticoid deficiency (2). Other causes of hypoadrenocorticism include infiltrative diseases such as neoplasia and fungal disease, and the administration of drugs for treating hyperadrenocorticism (3, 4). Dogs with hypoadrenocorticism show the age of onset with a range of 2 to 6 years, and certain breeds are predisposed for hypoadrenocorticism including Great Dane, Border terrier, Poodle, Rottweiler, West Highland white terrier, Basset hound, and Springer spaniel (5). Clinical signs of hypoadrenocorticism are non-specific including lethargy, weakness, anorexia, vomiting, and diarrhea (6). Treatment for hypoadrenocorticism include restoring tissue perfusion with correcting hypovolemia and electrolyte abnormalities such as hyponatremia and hyperkalemia (1, 7).

Hyponatremia is the most common electrolyte imbalance encountered in clinical practice (8). The severity of hyponatremia is known as to be related to a fatality rate in dogs and cats (9). Symptoms from hyponatremia might vary from subtle to severe or even life threatening, so it is important to correct severe hyponatremia immediately (8, 10). However, increasing serum sodium level abruptly can cause neurologic complications such as osmotic demyelination syndrome (ODS) and oculomotor abnormalities in humans and dogs (1, 2, 11).

This case report describes the clinical manifestations and outcome of hindlimb ataxia and seizures caused by rapid correction of hyponatremia in a dog presumed to have ODS concurrent with primary hypoadrenocorticism. To the author's knowledge, this is a rare case report that has shown hindlimb ataxia and seizures induced by presumed ODS in a dog with primary hypoadrenocorticism.

## Case Description

### Case Presentation and Diagnostic Investigations

A 6-year-old castrated male Chihuahua dog was referred for evaluation of clinical signs including hindlimb paresis and ataxia that developed after the correction of hyponatremia by the referring veterinarian.

Ten days previously, the dog had a history of vomiting and diarrhea, and hyponatremia (119.8 mmol/L; reference range, 144–160 mmol/L) with the ratio of sodium concentration to potassium concentration of 22.2 (Table 1, Day 0). The dog was hospitalized for a day and treated with 0.9% NaCl fluid therapy at a rate of 0.56 mEq/L/h for the correction of hyponatremia. An adrenocorticotropin hormone (ACTH; Synacthen; Dalim Bio Tech, Korea) stimulation test showed decreased cortisol concentration both before and after ACTH stimulation (pre-stimulation, <1 μg/dL; reference range, 1–6 μg/dL; post-stimulation, <1 μg/dL; reference range, 5.5–18 μg/dL), compatible with hypoadrenocorticism. Further, Administrations of prednisolone (0.25 mg/kg PO, BID; Yuhan, Korea), dexamethasone (0.25 mg/kg, IV; Jeilpharm, Korea), and desoxycorticosterone pivalate (DOCP, 2.2 mg/kg, IM; Elanco, USA) were initiated. However, 2 days after discharge, the dog developed five episodes of generalized tonic seizures, hindlimb paresis, and ataxia. Phenobarbital (2 mg/kg, PO, BID; Hanapharm, Korea) was initiated for the control of seizures, and magnetic resonance imaging (MRI) of the brain was performed for evaluation of the dog's neurologic status the day after onset of the neurological signs. However, the MRI findings were not remarkable, and the dog was referred to the Konkuk Veterinary Teaching Hospital.

**Table 1:** Complete blood count, serum biochemistry, and serum electrolytes results in a dog with primary hypoadrenocorticism and osmotic demyelination syndrome.

**Parameters**	**D0**	**D1**	**D6**	D10^*^	**D28**	**D78**	**Reference interval**
WBC (10^9^/L)	23.24	–	–	20.25	7.87	8.41	5.05–16.7
RBC (10^12^/L)	5.87	–	–	3.87	5.15	5.92	5.65–8.87
HCT (%)	38.3	–	–	25	36	40.7	37.3–61.7
Plt (10^3^/μL)	347	–	–	320	375	419	148–484
ALT (U/dL)	99	–	–	103	436	76	10–100
Na (mmol/L)	119.8	131.7	146	146	147	150	144–160
Cl (mmol/L)	91	103.3	106	108	108	110	109–122
K (mmol/L)	5.39	4.09	3.4	3.9	4.3	4.5	3.5–5.8
BUN (mg/dL)	50.5	12	–	10	16	10	7–27
Glucose (mg/dL)	141	–	–	116	103	106	74–143
Osmolality (mmol/kg)^†^	265.47	–	–	302.02	305.44	309.46	280–310

* *First visit at the hospital*.

† *Calculated: 2 × Na + Glucose/18 + BUN/2.8 (25)*.

*ALT, alanine transaminase; BUN, blood urea nitrogen; Cl, chloride; D, days after rapid correction of hyponatremia; HCT, hematocrit; K, potassium; Na, sodium; Plt, platelet; RBC, red blood cell; WBC, white blood cell*.

On physical examination, the dog had hindlimb paresis and ataxia. A neurologic examination revealed absence of right hindlimb proprioception and increased hindlimb spinal reflexes including patellar, gastrocnemius, and cranial tibial reflexes. Hindlimb crossed extensor was also shown, indicating hindlimb upper motor neuron signs. The rest of the neurological examination was unremarkable. Considering the history of seizures, neurologic localization included a diffuse forebrain and T3–L3 spinal segment.

The complete blood count revealed non-regenerative normochromic normocytic anemia with the reticulocyte index of 0.89 (HCT 25%; reference range, 37.3–61.7%) and leukocytosis (WBC 20.25 × 10^9^/L; reference range, 5.05–16.76 × 10^9^/L). Serum biochemistry profile presented mild elevation of alanine transaminase (103 U/dL; reference range, 10–100 U/dL) and serum electrolytes showed mild hypochloridaemia (108 mmol/L; reference range, 109–122 mmol/L; Table 1, Day 10). An ACTH stimulation was performed while administration of prednisolone was continued. The ACTH stimulation test showed that cortisol (pre-stimulation, 1.6; reference range, 1–6 μg/dL; post-stimulation, < 0.5; reference range, 5.5–18 μg/dL) and aldosterone (pre-stimulation, 0.1 ng/dL; reference range, 0.5–34.5 ng/dL; post-stimulation, 0.1 ng/dL; reference range; 3.6–21.6 ng/dL) were decreased. Assessment of plasma renin activity revealed increased plasma renin activity (8.69 ng/mL/h; reference range, 0.6–4.3 ng/mL/h), indicating primary hypoadrenocorticism.

Differential diagnosis for the neurologic signs included metabolic disease (anemia and thiamine deficiency), T3–L3 intervertebral disc degeneration (IVDD), and ODS. Considering the fact that the dog had a history of rapid correction of hyponatremia followed by neurologic signs developing 3 days after initial therapy, ODS was highly suspected. However, additional MRI assessment for definite diagnosis could not be performed because further evaluation was denied by the owner.

### Treatment and Outcome

Phenobarbital (2 mg/kg, PO, BID; Hanapharm, Korea) and prednisolone (0.25 mg/kg, PO, BID; Yuhan, Korea) with DOCP (2.2 mg/kg, IM, every 25 days; Elanco, USA) were continued to control seizures and primary hypoadrenocorticism. Other supportive medications including thiamine (2 mg/kg, PO, SID; Sinilpharm, Korea), vitamin B&C complex (0.5 T/divided, PO, BID; Yuhan, Korea), famotidine (0.5 mg/kg, PO, BID; Nelson, Korea), and zentonil (0.1 T/divided, PO, BID; Vetoquinol, USA) were initiated. Darbepoietin (0.45 μg/kg, SC; Kyowa hakko kirin, Korea) was administered for nonregenerative anemia induced by hypoadrenocorticism. The dog's gait was gradually normalized within 2 weeks and other additional clinical signs were not found. Moreover, phenobarbital was slowly tapered within 3 months, and no additional seizures occurred after drug was discontinued. The dog continued to be free of neurologic signs over the 19-month follow-up period.

## Discussion

The ODS, also called myelinolysis, is caused by rapid elevation of serum sodium inducing rapid fluid loss from brain cells and osmotic shrinkage of axons, which tears connection with myelin sheaths (10, 12). It is commonly observed in humans with rapid correction of hyponatremia, alcoholism, malnutrition, and liver disease (13–15), and neurologic symptoms can occur several days after fluid resuscitation in humans and dogs (1, 2, 16). Onset of clinical signs by ODS have been reported 2 to 6 days after rapid correction of hyponatremia in dogs (17–19), and a wider range of 1 to 14 days in humans (12). In case of dogs with ODS, neurologic clinical signs such as limb paresis, dysphagia, ataxia, and disorientation were revealed (17, 18, 20). ODS can be classified from two groups, central pontine myelinolysis (CPM) and extrapontine myelinolysis (EPM), and CPM is a demyelinating condition affecting principally the pons (21). Otherwise, lesions can occur outside the pons, such as cerebellum, hippocampus, cerebral cortex, and thalamus, so-called EPM (12). The pathological changes of both two groups are equal but they differ in clinical manifestations (12). Clinical signs of CPM include dysphagia, tetraparesis, and oculomotor abnormalities, otherwise dystonia, catatonia and behavioral changes are shown in EPM (1, 13).

In this case, the dog had clinical signs involving generalized seizures, hindlimb ataxia, and paresis developing 3 days after rapid correction of hyponatremia. In humans, seizures are one of the clinical signs caused by ODS (14, 21). In veterinary medicine, one canine case study reported that ODS induced whole-body spasms (18), which are automatic jerking movement belonging to a type of seizure (22, 23). Prognosis of ODS is variable, and full recovery of neurologic function after ODS could occur with supportive therapy (11, 14, 24). One human study reported that approximately two third of children affected by ODS were fully recovered from neurologic signs (21). In this case, neurologic clinical signs of the dog were gradually resolved, and after dropping anti-convulsant and supportive medication, the dog continued to be free of neurologic or additional clinical signs over the 19-month follow-up period.

ODS has characteristic MRI findings showing symmetrically increased signal intensity affecting brain lesions such as the pons, cerebellum, or hippocampus on T2-weighted images (13). However, ODS might not be detectable in MRI evaluation for up to 4 weeks (1, 13). It is reported that early MRI findings can be normal in 25% of cases in humans with ODS (13). Moreover, in a previous report (19), among 6 dogs showing neurologic signs with ODS experimentally induced by hyponatremia, 3 dogs (50%) had no imaging abnormalities. Therefore, a serial brain imaging is recommended for dogs with strongly clinical suspicion of ODS, and although there is no lesion on the MRI findings, ODS might not be excluded. In this case, the diagnosis of ODS could not be proven by MRI findings of the dog's brain. However, considering the facts that the owner stated that the dog had no history of any neurologic signs before the rapid correction of hyponatremia, and there were no additional neurologic signs after stopping anticonvulsant therapy, presumptive diagnosis of ODS secondary to rapid correction of hyponatremia was made in this dog.

Although less likely, thiamine deficiency and anemia can also cause nervous system problems in dogs. This dog did not have severe anemia and the neurological symptoms due to thiamine deficiency did not completely match the dog's clinical signs, so both causes were excluded. However, supportive thiamine and darbepoietin were used as adjunctive treatment to improve the dog's clinical signs. Additionally, T3–L3 intervertebral disc degeneration (IVDD) might be considered as differential diagnosis considering hindlimb ataxia and upper motor neuron signs. However, based on the absence of spinal hyperesthesia and resolution of clinical symptoms without specific treatment for intervertebral disc disease, it was unlikely that IVDD was a major cause of neurological symptoms of the dog.

To prevent ODS, it is recommended avoiding mineralocorticoids with fluid therapy at the same time because these could facilitate rapid increase of sodium level (1). Moreover, correction of hyponatremia should be limited to < 10 mmol/L over 24 h (8, 10) and be increased no faster than 0.5 mEq/L/h (1). In the case of severe malnutrition or hepatic disease, hyponatremia should be corrected at a rate of < 10 mEq/L in the first 24 h (20). When raising blood sodium level using fluid therapy, serum sodium concentration should be closely monitored by checking sodium levels 6 and 12 h, and daily after initiation of correction, until the serum sodium concentration has stabilized (8). All possible causes of ODS after rapid correction of hyponatremia described above are applicable to this case. In this case, 11.9 mmol/L of sodium level was increased within 24 h with the correction rate of 0.59 mEq/L/h. Moreover, the dog got the fluid therapy with administration of mineralocorticoids by DOCP injection simultaneously.

In conclusions, this is a case report that describes the clinical management and favorable outcome of presumed ODS developed by aggressive correction of hyponatremia in a dog with primary hypoadrenocorticism. All clinical signs including seizures and hindlimb ataxia were improved about 3 weeks after the first onset of the signs. ODS should be included among the differential diagnoses in case of acute neurological symptoms after episodes of rapid correction of hyponatremia. Moreover, subsequent course and prognosis of ODS has been variable, so correction of hyponatremia should be carefully done especially in diseases which can cause electrolytic imbalance such as hypoadrenocorticism.

## Data Availability Statement

The original contributions presented in the study are included in the article/supplementary material, further inquiries can be directed to the corresponding author.

## Ethics Statement

Ethical review and approval was not required for the animal study because informed consent was obtained from the present owner of the dog for publication of this case report and any accompanying images.

## Author Contributions

G-WL was involved in case analysis and was responsible for writing the manuscript. M-HK and H-MP were involved in case analysis and reviewing the manuscript. H-MP was involved in the coordination of the case and was responsible for interpretation of results. All authors contributed to the article and approved the submitted version.

## Conflict of Interest

The authors declare that the research was conducted in the absence of any commercial or financial relationships that could be construed as a potential conflict of interest.

## Publisher's Note

All claims expressed in this article are solely those of the authors and do not necessarily represent those of their affiliated organizations, or those of the publisher, the editors and the reviewers. Any product that may be evaluated in this article, or claim that may be made by its manufacturer, is not guaranteed or endorsed by the publisher.
